# Characteristics of Gut Microbiome and Its Metabolites, Short-Chain Fatty Acids, in Children With Idiopathic Short Stature

**DOI:** 10.3389/fendo.2022.890200

**Published:** 2022-06-10

**Authors:** Lin Li, Lifen Chen, Yuanyan Yang, Junqi Wang, Li Guo, Jingjing An, Xiaoyu Ma, Wenli Lu, Yuan Xiao, Xinqiong Wang, Zhiya Dong

**Affiliations:** ^1^ Department of Pediatrics, Ruijin Hospital, School of Medicine, Shanghai Jiao Tong University, Shanghai, China; ^2^ Department of Molecular Medicine, University of Utah School of Medicine, Salt Lake City, UT, United States

**Keywords:** idiopathic short stature, gut microbiome, *Faecalibacterium*, short-chain fatty acids, 16S rRNA sequence

## Abstract

**Background:**

The gut microbiome is important for host nutrition and metabolism. Whether the gut microbiome under normal diet regulate human height remains to be addressed. Our study explored the possible relationship between gut microbiota, its metabolic products and the pathogenesis of idiopathic short stature disease (ISS) by comparing the gut microbiota between children with ISS and of normal height, and also the short-chain fatty acids (SCFAs) produced by the gut microbiota.

**Methods:**

The subjects of this study were 32 prepubescent children aged 4-8 years. The fecal microbial structure of the subjects was analyzed by 16S rRNA high-throughput sequencing technology. The concentrations of SCFAs in feces were determined by gas chromatography-mass spectrometry.

**Results:**

The richness of gut microbiota in ISS group was decreased, and the composition of gut microbiota was significantly different between ISS group and control group. The relative abundance of nine species including family Ruminococcaceae and genera *Faecalibacterium* and *Eubacterium*, in ISS group was significantly lower than that in control group (P<0.05). The relative abundance of 10 species, such as those belonging to genus *Parabacteroides* and genus *Clostridium*, in ISS group was significantly higher than that in control group (P<0.05). The concentration of total SCFAs and butyrate in ISS group was significantly lower than that in control group. The correlation analysis among different species, clinical indicators, and SCFAs showed that the relative abundance of family Ruminococcaceae and genera *Faecalibacterium* and *Eubacterium* was positively correlated with the standard deviation score of height. Furthermore, the concentrations of total SCFAs and butyrate were positively correlated with serum insulin-like growth factor 1 (IGF-1)-SDS. Disease prediction model constructed based on the bacteria who abundance differed between healthy children and ISS children exhibited high diagnostic value (AUC: 0.88).

**Conclusions:**

The composition of gut microbiota and the change in its metabolite levels may be related to ISS pathogenesis. Strains with increased or decreased specificity could be used as biomarkers to diagnose ISS.

## Introduction

Idiopathic short stature (ISS) is one of the most common causes of short stature in children, accounting for 60%–80% of cases (1), and the etiology of ISS may involve a variety of factors, including genetics, hormones, nutrition, and the environment (2). Currently, recombinant human growth hormone (rhGH) is the main treatment for ISS. However, the therapeutic effects of rhGH differ in patients with ISS (3, 4). This may be due to the heterogeneity of individual patients and their potential etiology. Therefore, studying the pathogenesis of ISS and identifying biomarkers for early diagnosis is of great importance for formulating a reasonable treatment plan and improving lifelong height.

The human gut microbiome is a complex and dynamic microbial community, consisting of 10–100 trillion microbes, which carry approximately 100 times more genes than the human genome (5). A large number of previous studies have stated the potential role of gut microbiota in different aspects of human health, including early life (6, 7), as well as specific diseases, such as cardiovascular disease, metabolic disorders, malnutrition, inflammatory bowel disease, neuropsychiatric disease, and cancer (8–12). Reduced host size due to altered gut microbiota have been reported in different animals but remain unclear in human (13–17).

Early evidence of the effect of gut microbiota on host growth and development originated from studies in *Drosophila*. The researchers found that the development of flies without colonized gut microbiota lagged behind conventionally reared flies (16). Schwarzer et al. (15) obtained similar results in mice. The growth rate and body weight of germ-free (GF) newborn mice were lower than those of conventional feeding newborn mice. Subsequent studies found that depletion of gut microbiota reduced bone growth in mice due to decreased production of its metabolites including SCFAs, and the latter reduced insulin-like growth factor 1 (IGF-1) (14, 17). An et al. using a mouse model reported that intestinal *Lactobacillus* and short-chain fatty acids (SCFAs) were related to the catch-up growth of small for gestational age pups of rats, and that gut microbiota may play a role in the catch-up growth of these pups (18). The results of the above animal experiments confirmed the critical role of the gut microbiota on host growth.

In human, dysregulated gut microbiota have been associated with many diseases. In children, undernutrition leads to short stature and altered gut microbiota play an important role in this. However, as to children with normal diet, there are no studies on the role of the gut microbiota in the pathogenesis of ISS in children. With ISS and healthy children as research objects, this research explores the correlation between the gut microbiota and the pathogenesis of idiopathic short stature and finds diagnostic markers by analyzing the fecal microbiota structure of the research subjects and measuring the metabolism of short-chain fatty acids (SCFAs) in feces.

## Materials and Methods

### Subjects

ISS patients who visited the pediatric outpatient department of Ruijin Hospital affiliated with Shanghai Jiao Tong University School of Medicine between October 2018 and February 2020 were enrolled with written consent. Patients were recruited based on the following criteria: (1) the height of pre-adolescent children aged 4-8 years was two standard deviations (SD) lower than the normal reference value of the same region, sex, age, and race; (2) normal birth weight and body length, annual growth rate of height < 5 cm, and delayed or normal bone age; (3) the peak values of growth hormone (GH) stimulation tests were all greater than 10 µg/L; and (4) no other malformations or organic diseases (chronic diseases of the bone, liver, kidney, heart, and lung), no serious psychological and emotional disorders. The control group consisted of healthy children from East China. Inclusion criteria: 4-8 years old, and height within the range of plus or minus one standard deviation (-1SD to +1SD) of the mean normal reference values of the same sex, age, region, and race. The exclusion criteria were as follows: (1) family history of familial short stature and constitutively delayed pubertal development or any other genetic disease; (2) suffering from congenital malformation, mental retardation, special facial features, obesity, or digestive tract diseases; (3) received antibiotic and probiotic intervention in the last three months. All the patients and healthy controls were from east China (with similar dietary habits) to minimize the impact of diet differences on gut microbiota.

This study was approved by the Ethics Review Committee of Ruijin Hospital affiliated to Shanghai Jiao Tong University School of Medicine and was conducted in accordance with the principles of the Helsinki Declaration. All participants and their guardians provided informed consent and signed informed consent forms (Project identification code: KY2020-121).

### Sample Selection

Stool samplers and sterile stool sampling tubes (Sarstedt, Germany) were used to collect fresh stool samples from the subjects. Each tube of stool sample weighed 5–10 g and was stored in a refrigerator at -80°C. Four to five milliliters of elbow venous blood was collected from the subjects, into a pro-coagulant vacuum vein collection, and placed in a 4°C refrigerator for 2 h. The blood samples were centrifuged at 1300g/min for 15 min. The serum was saved to sterile tubes and stored at -80°C until further use.

### Determination of Serum IGF-1 and NT Pro-CNP Concentrations

Serum IGF-1 concentration was detected using an IMMULITE2000 IGF-1 kit (L2KGF2, Siemens, UK), following the manufacturer’s instructions (19). IGF-I standard deviation scores were calculated using the laboratory reference data for a survey of normal children (20). The CNP-22 ELISA kit (Cat. NO: EKE-012-03 Phoenix Pharmaceuticals Inc., USA) was used to detect the serum NT-proBNP concentration, following the manufacturer’s instructions.

### Bacterial DNA Extraction From Feces and 16S rRNA Amplification and Sequencing

Fecal bacterial DNA was extracted using TIA Namp Stool DNA Kit (Invitrogen, California, USA) according to the manufacturer’s protocol, DNA concentration and integrity were determined using a Nanodrop ND 1,000 Spectrophotometer (Thermo Fisher Scientific, United States) and the quality of the extracted DNA was evaluated by 1.2% agarose gel electrophoresis, respectively. PCR amplification of the bacterial 16S rRNA genes V3–V4 region was performed using the forward primer 338F (5’- ACTCCTACGGGAGGCAGCA -3’) and the reverse primer 806R (5’- GGACTACHVGGGTWTCTAAT -3’). Thermal cycling consisted of initial denaturation at 98°C for 2 min, followed by 25 cycles consisting of denaturation at 98°C for 15 s, annealing at 53°C for 30 s, and extension at 72°C for 30 s, with a final extension of 5 min at 72°C. PCR products were purified with Vazyme VAHTS™ DNA Clean Beads (Vazyme, Shanghai, China) and quantified using a Quant-iT™ PicoGreen dsDNA Assay Kit (Invitrogen, United States). Sequencing libraries were prepared using Illumina’s TruSeq Nano DNA LT Library Prep Kit (Illumina, Delaware, USA), and sequenced on an Illumina Hiseq2500 platform. The sequencing service was provided by Personal Biotechnology Co., Ltd (Shanghai, China).

### Bioinformatics Analysis

Sequencing data analyses were mainly performed using QIIME2 (https://docs.qiime2.org/2019.4/citation/) (21) and R packages (v3.6.0). The raw sequence data were demultiplexed and quality filtered using the q2‐demux plugin followed by denoising with DADA2 (22). All amplicon sequence variants (ASVs) were aligned with mafft (23) (via q2‐alignment) and used to construct a phylogeny with fasttree2 (24) (via q2‐phylogeny). ASV-level alpha diversity indices, such as Chao1 richness estimator, were calculated using the ASV table in QIIME2, by Kruskal-Wallis test, and visualized as box plots. Wilcoxon rank-sum test was utilized to detect differentially enriched microbiota at genus between ISS and healthy controls groups (P < 0.05). Beta diversity analysis was performed to investigate the structural variation of microbial communities among different samples using Bray-Curtis metrics and visualized *via* principal coordinate analysis (PCoA) (25). Non-parametric permutational multivariate analysis of variance (PERMANOVA) was conducted for analyzing significant difference of microbiota structure between the two groups. LEfSe (Linear discriminant analysis effect size) was performed to detect differentially abundant taxa in both groups (26). The raw data were deposited into the NCBI Sequence Read Archive (SRA) database (accession number PRJNA819198, https://www.ncbi.nlm.nih.gov/Traces/study/?acc=PRJNA819198).

### Detection of Short Chain Fatty Acids in Feces

With appropriate dose of acetic acid, propionic acid, butyric acid, isobutyric acid, valeric acid, isovaleric acid(Sigma, Germany) and caproic acid(Aladdin, The United States), diethyl ether (Sinopagic Chemical Reagent Company, China) was added to make 10 mixed-standard concentration gradients(0.02 μg/mL, 0.1 μg/mL,0.5 μg/mL, 2 μg/mL,10 μg/mL,25 μg/mL,50 μg/mL,100 μg/mL,250 μg/mL,500 μg/mL). Feces were thawed on ice, and approximately 50 mg of feces were homogenized in 50 μl 15% phosphoric acid. The suspensions were homogenized with a vortex and centrifuged at 15285g for 10min at 4°C. The supernatants were processed for gas chromatography-mass spectrometry on a Thermo TRACE 1310-ISQ gas mass spectrometer (Thermo, Massachusetts, USA). The injection volume was 1 μl, and the split ratio was 10:1. Samples were separated with an Agilent HP-INNOWAX capillary GC column (30 m × 0.25 mm ID × 0.25 µm). The initial temperature was 90°C and was increased to 120°C at 10°C/min, after which the temperature was increased to 150°C at 5°C/min and then to 250°C at 25°C/min, where it remained for 2 min. The carrier gas was helium (1.0 ml/min). The temperatures of the injection port and ion source were 250°C and 230°C respectively under SIM model. Agilent MSD ChemStation software was used for quantitative analysis of chromatographic peak area and retention time (27).

### Data Analysis

Data documentation and statistical analyses were performed using SPSS software version 23.0. Parametric data are expressed as the mean and standard deviation (SD). SD scores were calculated for height (Ht SDS) and IGF-1 (IGF-1 SDS). The differences between the groups were examined using the independent sample t-test. Continuous nonparametric data are represented as median values (interquartile spacing [IQR]) and were analyzed using the Wilcoxon rank-sum test. Receiver operating characteristic (ROC) analysis was performed to assess the diagnostic value of the identified indicators by using pROC package and ggplot2 package of R language. Spearman rank coefficient was used to test the correlation between the two study variables. The correlation coefficient (r) > 0.3 and < 0.5 was considered to be moderate correlation. The correlation coefficient (r) > 0.5 was considered to be strongly correlated, and *P* < 0.05 was considered to be statistically significant.

## Results

### Clinical Features

**Figure 1:**
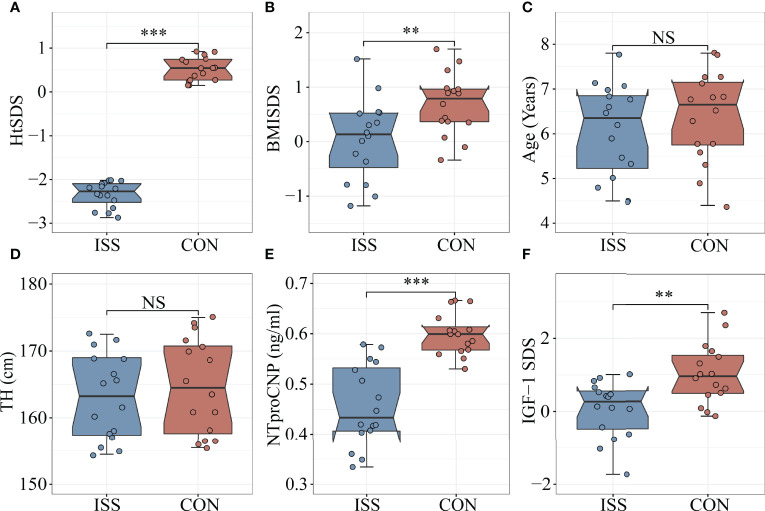
Comparison of biomarkers and Clinical features between ISS patients and healthy controls. **(A)** HtSDS; **(B)** BMI-SDS; **(C)** Age; **(D)** Target height; **(E)** NTproCNP; **(F)** IGF-1 SDS. **P <0.01; ***P < 0.001. HtSDS, height standard deviation score; BMI SDS, body mass index standard deviation score; TH, target height; NTproCNP, amino-terminal propeptide of C-type natriuretic peptide; IGF-1 SDS, insulin like growth factor standard deviation score. NS, no significance.

Thirty-two subjects aged 4-8 years were enrolled in the study. Sixteen patients with ISS (median age 6.4 years, 44% males) were enrolled as the ISS group and 16 healthy children of normal height (median age 6.6 years, 50% males) were enrolled as the control group. The height standard deviation score (HtSDS) of the ISS group was -2.33 ± 0.29, while that of the control group was 0.52 ± 0.27, with a statistical difference between the two groups (*P* < 0.001) (**Figure 1A**). The body mass index standard deviation score (BMI SDS) of the ISS group was 0.04 ± 0.74, and the BMI SDS of the control group was 0.69 ± 0.56. There was a statistically significant difference between the two groups (*P* = 0.009) (**Figure 1B**). Age, sex, and target height (TH) did not significantly differ between the ISS and control groups (*P* > 0.05) (**Figures 1C, D**). The GH peak of the growth hormone stimulation test in the ISS group was 12.77 ± 1.9 ng/mL.

### The Longitudinal Growth Regulatory Factors Decreased Significantly in ISS Patients

As mentioned, IGF-1 and amino-terminal propeptide of C-type natriuretic peptide (NTproCNP) deficiencies are known factors contributing to the short stature. To evaluate the growth of children with ISS and healthy controls, we measured the levels of IGF-1 and NTproCNP, which are important regulators of serum longitudinal bone growth in childhood. The results showed that the serum NTproCNP of ISS group was 0.46 ± 0.08 ng/mL, and that of the control group was 0.60 ± 0.04 ng/mL. There was a statistically significant difference between the two groups (*P* < 0.001) (**Figure 1E**). The integral standard deviation of IGF-1 (IGF-1 SDS) of ISS group was 0.06 ± 0.77, and that of control group was 1.03 ± 0.82, indicating a statistically significant difference between the two groups (*P* = 0.002) (**Figure 1F**).

### Gut Microbiota of ISS Patients

**Figure 2:**
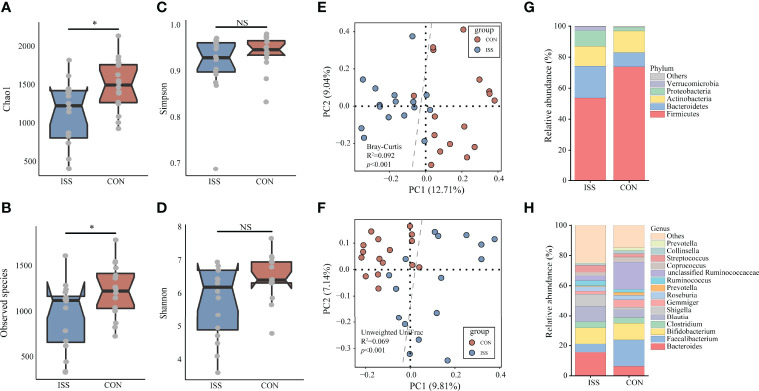
The α-diversity of the microbiota, presented as the Chao1 index **(A)**, Observed species index **(B)**, Simpson index **(C)**, and Shannon index **(D)**, from ISS patients and healthy subjects was calculated (Chao1 index, *P* = 0.018; Observed species index, *P* = 0.038). The gut microbiota composition of ISS patients was significantly different from that of healthy subjects. The β-diversity of microbiota in the ISS and CON groups was calculated by the Bray-Curtis distance **(E)** and unweighted UniFrac distance **(F)** and is shown in the PCoA plot (Adonis test, Bray-Curtis distance, R2 = 0.092, *P* < 0.001; unweighted UniFrac distance, R2 = 0.069, *P* < 0.001). Each point represents the composition of the microbiota of one participant. The boxplots display the predominant bacterial taxa at the phylum **(G)** and genus **(H)** levels in the ISS and CON groups. *P < 0.05. NS, no significance.

We next evaluated the gut microbiota in ISS, as evidence from animal studies suggests that gut microbiota can regulate host growth (15, 16, 18). We collected stool samples from our ISS patients and control cohort for 16S rRNA profiling. The alpha diversity index was used to analyze the richness and diversity of gut microbiota. The analysis results showed that Chao1 index and Observed species index in ISS group were significantly lower than those in control group (*P* = 0.018, *P* = 0.038), suggesting that the species richness of ISS group was lower than that of control group (**Figures 2A, B**). Simpson and Shannon indices, reflecting the diversity between the two groups, showed no significant differences (**Figure 2**).

Beta diversity analysis showed that the gut microbiota of the ISS and control groups were significantly separated, and the dispersion degree between samples was large; however, gut microbiota among samples from the same group appeared to be relatively concentrated. Adonis analysis was used to verify the difference between the two groups. The results confirmed that there were significant differences in gut microbiota between the two groups (Adonis test, Bray-Curtis distance, R2 = 0.092, *P* < 0.001; unweighted UniFrac distance, R2 = 0.069, *P* < 0.001), indicating significant changes in the composition of gut microbiota in ISS patients (**Figures 2E, F**). The main strains at the phylum and genus levels in the ISS and control groups are shown in **Figures 2G, H**. The main strains at the phylum level were Firmicutes, Bacteroidetes, Actinobacteria, Proteobacteria, and Verrucomicrobia. The main strains at the genus level were *Bacteroides*, *Faecalibacterium*, *Bifidobacterium*, *Clostridium*, and *Blautia*.

**Figure 3:**
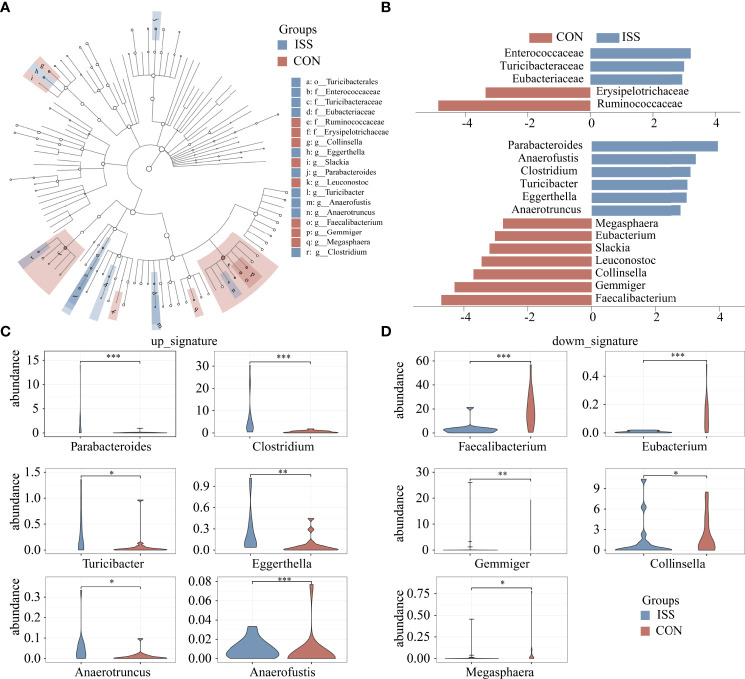
Cladogram based on LEfSe results of the CON and ISS groups. The blue points represent the increased taxa in ISS group, while the red points represent the increased taxa in CON group **(A)**. **(B)** Linear discriminant analysis effect size (LEfSe) algorithm of intestinal microbiota at the family and genus levels (LDA > 2.5). **(C, D)** Violin plots depict the relative abundances of the top 11 discriminatory species in the ISS and control groups. ISS, Idiopathic short stature; CON, healthy subjects serving as controls. *P < 0.05; **P < 0.01; ***P < 0.001.

Linear discriminant analysis effect size (LEfSe) was used to analyze the difference in abundance of species between the two groups and to determine which species contributed to the significant difference between groups. Five different species were screened at the family level, as shown in **Figure 3A**. Compared to the control group, the relative abundance of Ruminococcaceae and Erysipelotrichaceae in the ISS group was significantly decreased, while the relative abundance of Enterococcaceae, Turicibacteraceae, and Eubacteriaceae was significantly increased in ISS group. Thirteen different species were found significantly different at the genus level. Compared to the control group, the relative abundance of *Faecalibacterium*, *Eubacterium*, *Gemmiger*, *Collinsella*, *Leuconostoc*, *Slackia*, and *Macroglobules* was significantly decreased, while the relative abundance of *Parabacteroides*, *Clostridium*, *Anaerofustis*, *Turicibacter*, *Eggerthella*, and *Anaerotruncus* was significantly increased in the ISS group. **Figure 3B** shows the results of LDA Effect Size of species in the ISS group and control group at the family and genus levels. **Figure 3C** shows the increased genus of bacteria in ISS and **Figure 3D** the decreased bacteria in ISS. Of these, The genus *Faecalibacterium* is one of the most abundant and important symbiotic bacteria in the human gut microbiota, accounting for approximately 5% of the total bacteria in feces and belongs to the family Ruminococcaceae (28, 29). The genus *Eubacterium* is also one of the core genera of the human gut microbiota, which is widely found in the human intestinal tract and has high specificity and adaptability to the human intestinal tract (30, 31).

### SCFA Levels in Feces of ISS Patients Decreased

**Figure 4:**
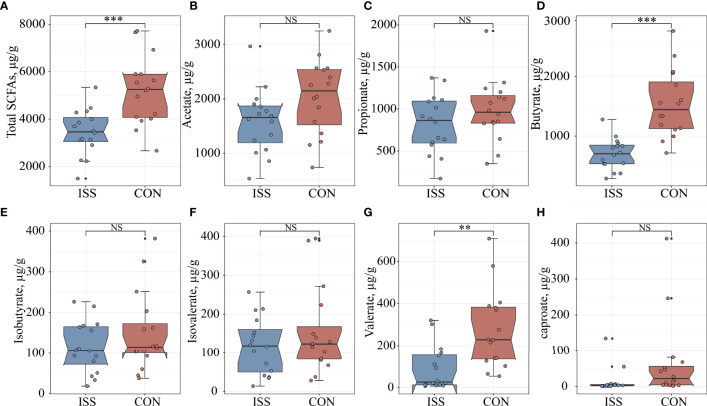
Comparison of seven fecal SCFA levels between ISS patients and healthy controls. **(A)** total SCFAs; **(B)** acetate; **(C)** propionate; **(D)** butyrate; **(E)** isobutyrate; **(F)** isovalerate; **(G)** valerate; **(H)** caproate. **P < 0.01; ***P < 0.001. NS, no significance.

**Figure 5:**
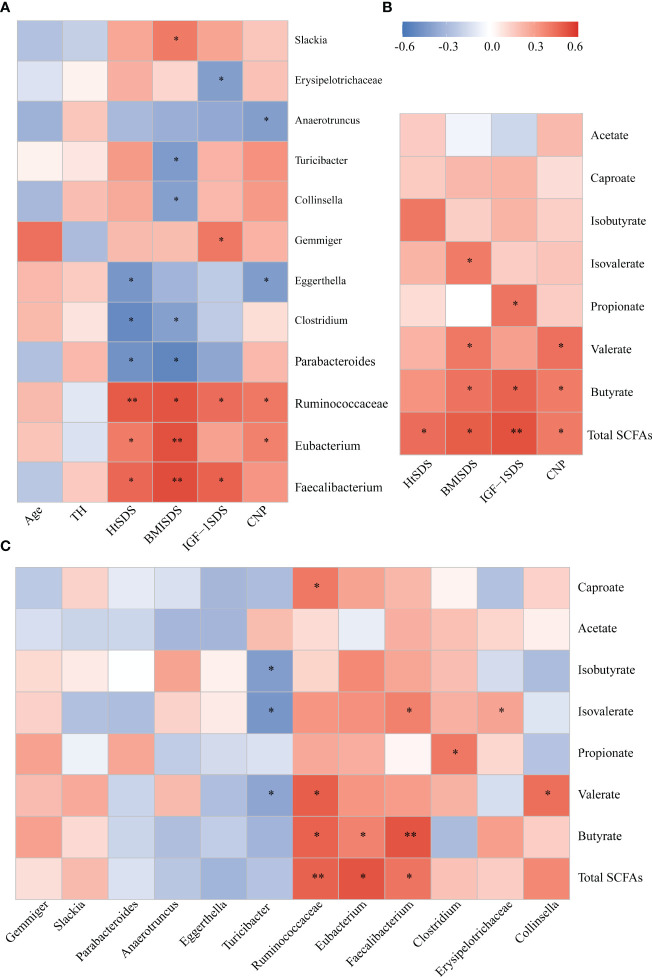
**(A)** Associations of gut microbial taxa with clinical indexes. Heat map of Spearman’s rank correlation coefficient among six clinical indexes and 12 taxa; **(B)** A heatmap of Spearman correlation between clinical indexes and SCFA; **(C)** A heatmap of Spearman correlation between gut microbiota and SCFAs. Red: positive correlation; blue: negative correlation; *P < 0.05, **P < 0.01. SCFAs, short-chain fatty acids.

We measured if ISS is associated with altered SCFA levels in our patient cohort. Quantitative analysis of SCFAs concentrations in fecal samples by GC-MS showed significant differences between the ISS and control group. The total SCFAs concentration of ISS group was 3450.33 ± 963.94 µg/g; butyrate: 698.35 ± 261.96 µg/g; valerate: 91.98 ± 105.79 µg/g. The total SCFA concentration in the control group was 5200.43 ± 1440.66 µg/g; butyrate, 1534.99 ± 563.71 µg/g; valerate: 275.80 ± 186.08 µg/g. The concentrations of total SCFAs and individual components butyrate and valerate in the ISS group were significantly lower than those in the control group (*P* < 0.05) (**Figure 4**). Spearman correlation analysis was carried out between SCFAs and serum bone longitudinal growth regulatory factors, and the results showed that total SCFA and butyrate levels were significantly positively correlated with IGF-1 SDS levels (R > 0.4, *P* < 0.05) (**Figure 5B**).

### Correlation Analysis of Different Strains, Clinical Indicators, and SCFAs

Spearman correlation analysis was conducted between different bacterial species and clinical indicators to determine the correlation between gut microbiota composition and host health status (**Figure 5A**). The family Ruminococcaceae and genera *Faecalibacterium* and *Eubacterium* were significantly positively correlated with HtSDS (r = 0.502, *P* = 0.005; r = 0.466, *P* = 0.047; r = 0.405, *P* = 0.023, respectively), suggesting that their deficiency exerts an influence on the development of ISS. In contrast, family Ruminococcaceae was positively correlated with serum levels of NTproBNP (r = 0.414, *P* = 0.028). The genera *Parabacteroides* and *Clostridium* were negatively correlated with HtSDS (r = -0.468, *P* = 0.045; r = -0.500, *P* = 0.047, respectively).

To evaluate whether the altered abundancy of gut Ruminococcaceae and genera *Faecalibacterium* and *Eubacterium* and others regulates the SCFAs in the host, Spearman correlation analysis was conducted between different bacterial species and SCFA levels (**Figure 5C**), and the results showed that the family Ruminococcaceae and genera *Faecalibacterium* and *Eubacterium* were significantly positively correlated with the concentrations of total SCFAs and butyrate (0.6> r > 0.4, *P* < 0.05). These results suggest that the significant decrease in SCFA concentration in the ISS intestine is closely related to the changes in intestinal microflora function.

### Receiver Operating Characteristic Curve (ROC) Analysis of Different Strains and Metabolites

**Figure 6:**
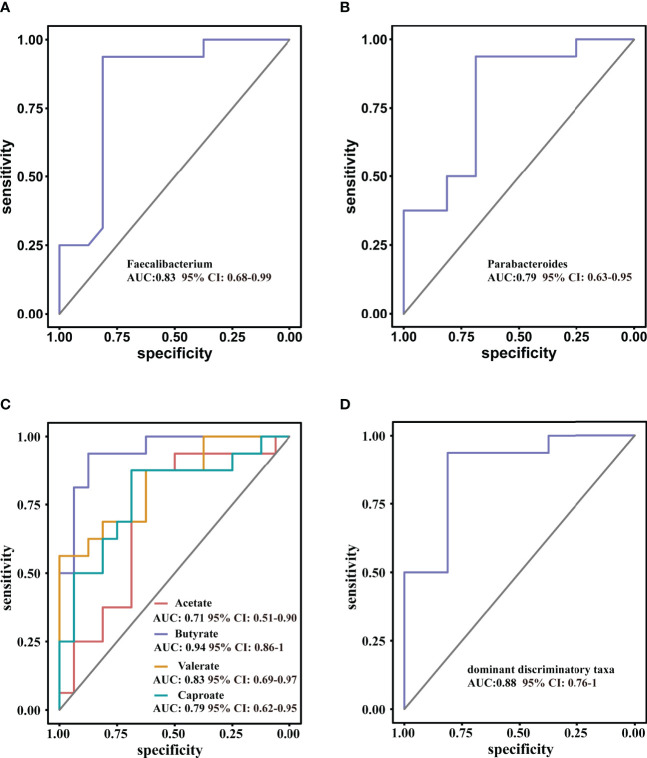
Receiver operating characteristic curve (ROC) analysis for diagnostic indicators **(A, B)** the predominant bacterial taxa that may be used to distinguish ISS from CON. **(C)** ROC curve of the model using four discriminatory metabolites; butyrate has high diagnostic value in discriminating ISS patients from CON. **(D)** ROC curve of the model using two discriminatory taxa.

ROC curve analysis was used to further evaluate the differential bacteria and metabolites to explore their diagnostic value for ISS. The area under the curve (AUC) for genus *Faecalibacterium*, which showed the most significant reduction in relative abundance in ISS group, was 0.83 (95% CI: 0.68–0.99, *P* = 0.001), and the cut-off value was 0.60 (sensitivity: 0.94, specificity: 0.78) (**Figure 6A**). The AUC of genus *Parabacteroides*, which showed the most significant increment in relative abundance in ISS group, was 0.79 (95% CI: 0.63–0.95, P =0.005). The cut-off value was 0.38 (sensitivity: 0.89, specificity: 0.68) (**Figure 6B**). We further included both genera *Faecalibacterium* and *Parabacteroides* as indices for binomial logistic regression analysis to construct a joint diagnostic model. As shown in **Figure 6D**, the AUC value, sensitivity, and specificity of the diagnostic model were higher: AUC value was 0.88 (95% CI: 0.76–1, *P* < 0.001), and cut value was 0.55 (sensitivity: 0.94, specificity: 0.81), indicating an improved diagnostic model.

The diagnostic value of the metabolites in ISS was evaluated, as shown in **Figure 6C**. The AUC value of acetate was 0.70 (95% CI: 0.52–0.92, *P* = 0.46) and the cut-off value was 2006.67 (sensitivity: 0.88, specificity: 0.63). The AUC value of butyrate was 0.94 (95%CI: 0.86–0.99, *P* < 0.001), and the cut-off value was 994.42 (sensitivity: 0.94, specificity: 0.88). The AUC value of valerate was 0.83 (95% CI: 0.69–0.97, *P* = 0.001) and the cut-off value was 42.51 (sensitivity: 0.56, specificity: 0.82). The AUC value of caproate was 0.76 (95% CI: 0.62–0.95, *P* = 0.006), and the cut-off value was 9.24 (sensitivity: 0.88, specificity: 0.69). These results indicate that butyrate has a high diagnostic value and could be used as a reference in clinical practice.

## Discussion

ISS is a major reason of short stature in children and affects children’s physical and mental health. However, the etiology of ISS is highly heterogeneous, and some patients with ISS do not respond to rhGH treatment. The exploration of its pathogenesis and the search for early diagnostic markers are still the focus of research.

This study included children with ISS and healthy children of normal height in East China as research objects, and analyzed and compared the composition of gut microbiota between the two groups. The results showed that the gut microbiota richness of the ISS group was lower than that of the control group. There were significant differences in the composition of gut microbiota between the ISS and control groups, indicating that the composition of gut microbiota in children with ISS changed significantly. Bacterial communities of the two groups were further analyzed and compared using LEfSe, and the differences between the two groups were found, mainly in *Faecalibacterium*, *Eubacterium*, *Gemmiger*, *Collinsella*, *Slackia*, *Parabacteroides*, *Clostridium*, *Turicibacter*, *Eggerthella* and *Anaerotruncus* genera. The relative abundance of the family Ruminococcaceae and genera *Faecalibacterium* and *Eubacterium* were significantly positively correlated with HT-SDS and BMI-SDS. Analysis of SCFAs from intestinal microbiota showed that the concentrations of total SCFAs and individual components, including butyrate, in the ISS group were significantly lower. Differential metabolites were positively correlated with the regulatory factors of longitudinal bone growth in children. These results suggest that changes in gut microbiota may be associated with the growth and development of ISS.

These bacteria are major components of the gut microbiome. As mentioned, the genus *Faecalibacterium*, a member of the Ruminococcaceae family, is one of the most abundant and important symbiotic bacteria in the human gut microbiota (28, 29). It is an important indicator of intestinal health and maintenance of intestinal homeostasis and decreased *Faecalibacterium* have been associated with inflammatory bowel disease, and diabetes diseases (32). The genus *Eubacterium* is also one of the core genera of the human gut microbiota, which is widely found in the human intestinal tract and has high specificity and adaptability to the human intestinal tract (30, 31). Consensus of intestinal microbial studies shows that the genera *Faecalibacterium* and *Eubacterium* are the main producers of SCFAs, including butyrate, in the intestinal tract (33), and like Bifidobacteria and other strains are considered beneficial to human health (34). Commercially, probiotics with Faecalibacterium supplementals are available and may have therapeutic potential in ISS.

Dysregulated gut microbiome is associated with decreased IGF-1 in our study. The role of IGF-1 in regulating bone development and promoting growth has been well demonstrated. A number of studies have confirmed that gut microbiota is closely correlated with IGF-1, and gut microbiota affects the levels of IGF-1 and its homologous genes in *Drosophila*, zebrafish, and mice (13, 35, 36). The mechanism by which the gut microbiota affects IGF-1 levels in the host remains unclear, but decreased SCFAs are considered as an important mechanism. In the growth hormone stimulation test of children with ISS in our study, the growth hormone peak was at a normal level. Although IGF-1 is downstream of growth hormone, the data showed that the effect of gut microbiota on IGF-1 was not regulated by changes in growth hormone levels alone. In the study by Yan et al. (17) depletion of gut microbiota does not change the growth hormone levels in mice, but reduced the IGF-1 production by downregulation of the receptor activator of nuclear factor-kappaB ligand (Rankl) expression. RANKL is the master regulator of osteoclastogenesis. One of the mechanisms how gut microbiota regulates RANKL and serum IGF-1 levels may be through the generation of SCFAs (14, 17).

Our study also found a positive correlation between SCFAs, including butyrate, and serum IGF-1 in the feces of children with ISS. Studies in mouse models suggest that IGF-1 may be an important factor mediating the production of intestinal microflora metabolites and the host growth (17). SCFAs are produced by the gut microbiota through fermentation of non-digestible carbohydrates (NDCs), mainly including acetate, propionate, and butyrate. SCFAs provide energy sources for the growth of intestinal microorganisms and various physiological effects of the host, and have also been confirmed to affect glucose and lipid metabolism, immunity, inflammation, and growth of the host. Butyrate plays a key role in intestinal health (37–40). Although butyrate has not been studied in ISS, it is critical for host energy metabolism, and bone formation (41, 42). In the study by Tyagi and colleagues, dietary supplemental of butyrate in mice increased the expansion of bone marrow regulatory T cells and promoted bone formation and bone mass acquisition (43). It would be interesting to further evaluate the butyrate level and regulatory T cells in future ISS studies.

The family Ruminococcaceae and genera *Faecalibacterium* and *Eubacterium*, whose abundance was significantly reduced in ISS patients in our study, are the main bacterial species producing SCFAs, especially butyrate, in the intestinal tract (33, 44, 45). Disrupted flora may negatively affect the supply of SCFAs, including butyrate, to intestinal epithelial cells. This study confirmed that the contents of SCFAs, especially butyrate, in the feces of ISS patients were significantly lower than those in the control group. Correlation analysis showed that the concentrations of total SCFAs and butyrate were significantly and positively correlated with IGF-1 SDS. These results suggest that there is a relationship between gut microbiota, SCFAs, and serum IGF-1 levels in patients with ISS. Studies have shown that SCFA levels in the intestinal tract of conventionally raised mice are higher than those in germ-free newborn mice (46). Germ-free mice showed an increase in intestinal SCFAs concentrations after routine colonization, as expected. In Yan J’s study, broad-spectrum antibiotics or vancomycin reduced intestinal SCFAs content and serum IGF-1 concentration in conventionally fed mice. To test whether SCFAs supplementation is sufficient to increase the circulating IGF-1 concentration in antibiotic-treated mice, SCFAs were added to drinking water. The serum IGF-1 level in antibiotic-treated mice supplemented with SCFAs was higher than that in the control group (17). These animal studies support the link between SCFAs, metabolites of the gut microbiota, and IGF-1.

Interestingly, in the absence of any gut microbiota, SCFA supplementation alone is not sufficient to fully restore the bone phenotype of the host (17), suggesting that there may be other mechanisms through which gut microbiota regulates host bone development (47, 48). Previous studies have shown that for many diseases affecting height, the level of serum NTproCNP in patients is consistent with the change in height or growth rate (49–51). Growth plate osteoblasts (52) and osteoclasts (53) express CNP and activate the guanosine cyclase/cyclic guanosine phosphate (GC-B/cGMP) signaling pathway in an autocrine and paracrine manner to promote the synthesis of cartilage matrix and stimulate the proliferation and differentiation of chondrocytes (54). At present, the regulation of serum CNP levels in tissues other than growth plates remains undiscovered. In this study, we found that the level of serum NTproCNP in the ISS group was significantly lower than that in the control group, which was related to the decrease in abundance of the family Ruminococcaceae. Therefore, gut microbiota is associated with circulating NTproCNP levels, which may affect growth and play a role in the pathogenesis of ISS.

At present, the etiology of ISS is unclear, and diagnosis is difficult. The improvement of medical history and physical examination should be accompanied by laboratory examination, imaging examination, genetic analysis, etc. The effect of growth hormone deficiency, genetic metabolic syndrome, organic disease, and short stature due to other secretory diseases was not included in this study. However, the economic values and duration of tests were relatively high. In this study, it was found that some specific bacteria increased or decreased in children with ISS. ROC curve analysis showed that the joint diagnostic model constructed using the genera *Faecalibacterium* and *Parabacteroides* as indices has a high value in differentiating ISS from healthy children. It plays a role in the early clinical identification of ISS. In addition, the high sensitivity and specificity of butyrate, a metabolite of the gut microbiota, may contribute to the diagnosis of ISS. It must be admitted that diet habits potentially affect the structure of the gut microbiota and may be the limitation of this study.

In conclusion, this study used high-throughput sequencing technology to analyze and compare the composition of gut microbiota in children with ISS and healthy children and found the genera *Faecalibacterium* and *Eubacterium*, and their metabolites, such as butyrate, are closely related to ISS. It indicated that the pathogenesis of ISS may be related to the composition of gut microbiota and short-chain fatty acid metabolism disorder. In this study, a diagnostic model was further established, which suggested that the genera *Faecalibacterium* and *Parabacteroides*, and butyrate have diagnostic value for ISS. This study provides an experimental basis for finding a more effective and safe diagnosis and treatment for patients with ISS.

## Data Availability Statement

The datasets presented in this study can be found in online repositories. The names of the repository/repositories and accession number(s) can be found below: NCBI [accession: PRJNA819198].

## Ethics Statement

The studies involving human participants were reviewed and approved by the Ethics Review Committee of Ruijin Hospital affiliated to Shanghai Jiao Tong University School of Medicine. Written informed consent to participate in this study was provided by the participants’ legal guardian/next of kin.

## Author Contributions

LL and LC contributed equally to this work. Conceptualization, LL, XW, and ZD. Data curation, LL and LC. Formal analysis, JA and XW. Methodology, LL and WL. Project administration, LL. Resources, YY, WL, YX, and ZD. Supervision, YX. Validation, LL. Writing – original draft, LL and LC. Writing – review & editing, LG, XW and ZD. All authors contributed to the article and approved the submitted version.

## Conflict of Interest

The authors declare that the research was conducted in the absence of any commercial or financial relationships that could be construed as a potential conflict of interest.

## Publisher’s Note

All claims expressed in this article are solely those of the authors and do not necessarily represent those of their affiliated organizations, or those of the publisher, the editors and the reviewers. Any product that may be evaluated in this article, or claim that may be made by its manufacturer, is not guaranteed or endorsed by the publisher.
